# The many faces of atrioventricular block in myocarditis: a case series and proposed clinical strategy highlighting cardiovascular magnetic resonance-guided diagnosis, device selection, and disease monitoring

**DOI:** 10.1093/ehjcr/ytag699

**Published:** 2026-09-15

**Authors:** Michiel J Bom, Luuk H G A Hopman, Vokko P van Halm, Suzan F M Nijman, Mariette Labots, Michel W P Tsang-A-Sjoe, Lourens F H J Robbers, P Stefan Biesbroek

**Affiliations:** Department of Cardiology, Amsterdam UMC, Vrije Universiteit Amsterdam, De Boelelaan 1118, 1081 HZ Amsterdam, The Netherlands; Department of Cardiology, Amsterdam UMC, Vrije Universiteit Amsterdam, De Boelelaan 1118, 1081 HZ Amsterdam, The Netherlands; Department of Cardiology, Amsterdam UMC, Vrije Universiteit Amsterdam, De Boelelaan 1118, 1081 HZ Amsterdam, The Netherlands; Department of Pulmonary Medicine, Amsterdam UMC, Vrije Universiteit Amsterdam, De Boelelaan 1118, 1081 HZ Amsterdam, The Netherlands; Department of Medical Oncology, Cancer Center Amsterdam, Amsterdam UMC, Vrije Universiteit Amsterdam, De Boelelaan 1118, 1081 HZ Amsterdam, the Netherlands; Department of Rheumatology and Clinical Immunology, Amsterdam UMC, Vrije Universiteit Amsterdam, De Boelelaan 1118, 1081 HZ Amsterdam, The Netherlands; Department of Cardiology, Amsterdam UMC, Vrije Universiteit Amsterdam, De Boelelaan 1118, 1081 HZ Amsterdam, The Netherlands; Department of Cardiology, Amsterdam UMC, Vrije Universiteit Amsterdam, De Boelelaan 1118, 1081 HZ Amsterdam, The Netherlands

**Keywords:** Cardiac magnetic resonance, Myocarditis, Atrioventricular conduction disorders, Atrioventricular block, Case series, Abatacept

## Abstract

**Background:**

Atrioventricular (AV) conduction disorders are a relatively rare complication in acute myocarditis. Although frequently transient in nature, persistent AV block has been reported. Cardiac magnetic resonance (CMR) imaging is critical in the diagnostic work-up and follow-up of myocarditis. This case series describes the use of CMR imaging to guide diagnosis, device selection, and disease monitoring.

**Case Summary:**

Four patients with complete AV block due to acute myocarditis underwent serial CMR imaging for diagnosis, disease monitoring, and device timing and selection. Recovery of AV conduction was variable in the presented cases. Cardiac magnetic resonance imaging was able to identify two distinct clinical scenarios of disease progress and subsequent AV conduction recovery: first, two patients (Cases 1 and 2) in which resolution of myocardial inflammation was paralleled by resolution of AV conduction disorders and, second, a patient (Case 3) in which AV block persisted even after resolution of inflammation due to the development of myocardial fibrosis. However, in one patient (Case 4), CMR was unable to adequately detect the disease progress, i.e. a patient with persistent AV block despite resolution of inflammation and no signs of myocardial fibrosis.

**Discussion:**

This case series illustrates the variable clinical course of AV conduction disorders in the context of acute myocarditis. Cardiac magnetic resonance imaging was essential in the identification of underlying myocarditis, offering insights in the disease course, and may therefore be used as an adjunctive tool for device selection. However, mismatch between CMR findings and AV conduction disorders may occur and warrants further investigation.

Learning points

- Although often transient in nature, the course of AV conduction disorders in the setting of myocarditis is highly variable and differs with different aetiologies.
- Cardiac magnetic resonance imaging is critical in identifying underlying myocarditis in at-risk patients, can be used as an adjunctive tool for disease monitoring, and may contribute to clinical decision-making regarding device therapy.
- Mismatch between CMR findings and AV conduction disorders may occur and warrants further investigation.

## Introduction

Acute myocarditis is an inflammatory disease of the myocardium most frequently triggered by a post-viral immune response. It may also occur in the context of systemic or auto-immune disease, either *de novo* as in systemic lupus erythematosus (SLE) or sarcoidosis or induced by immune checkpoint inhibitors (ICI), as well as after exposure to cardiotoxic agents such as chemotherapy. The clinical presentation of myocarditis is highly variable, ranging from mild symptoms to overt heart failure, cardiogenic shock, and ventricular arrythmias.^1^ Importantly, the disease course is often self-limiting, with partial or complete recovery of cardiac function.

Atrioventricular (AV) conduction disorders may complicate myocarditis.^2,3^ Similar to myocardial dysfunction, these conduction abnormalities are often reversible. Therefore, current guidelines advise to postpone permanent pacemaker implantation to wait for potential recovery.^4,5^

Cardiac magnetic resonance (CMR) imaging with tissue characterization using T2-weighted turbo spin echo (T2w-TSE), T1 and T2 mapping, and late gadolinium enhancement (LGE) is critical in the diagnostic work-up and follow-up of myocarditis.^5^ Cardiac magnetic resonance imaging may provide insight into the extent, distribution, and evolution of myocardial injury, potentially aiding in the prediction of conduction recovery.

**Figure 1:**
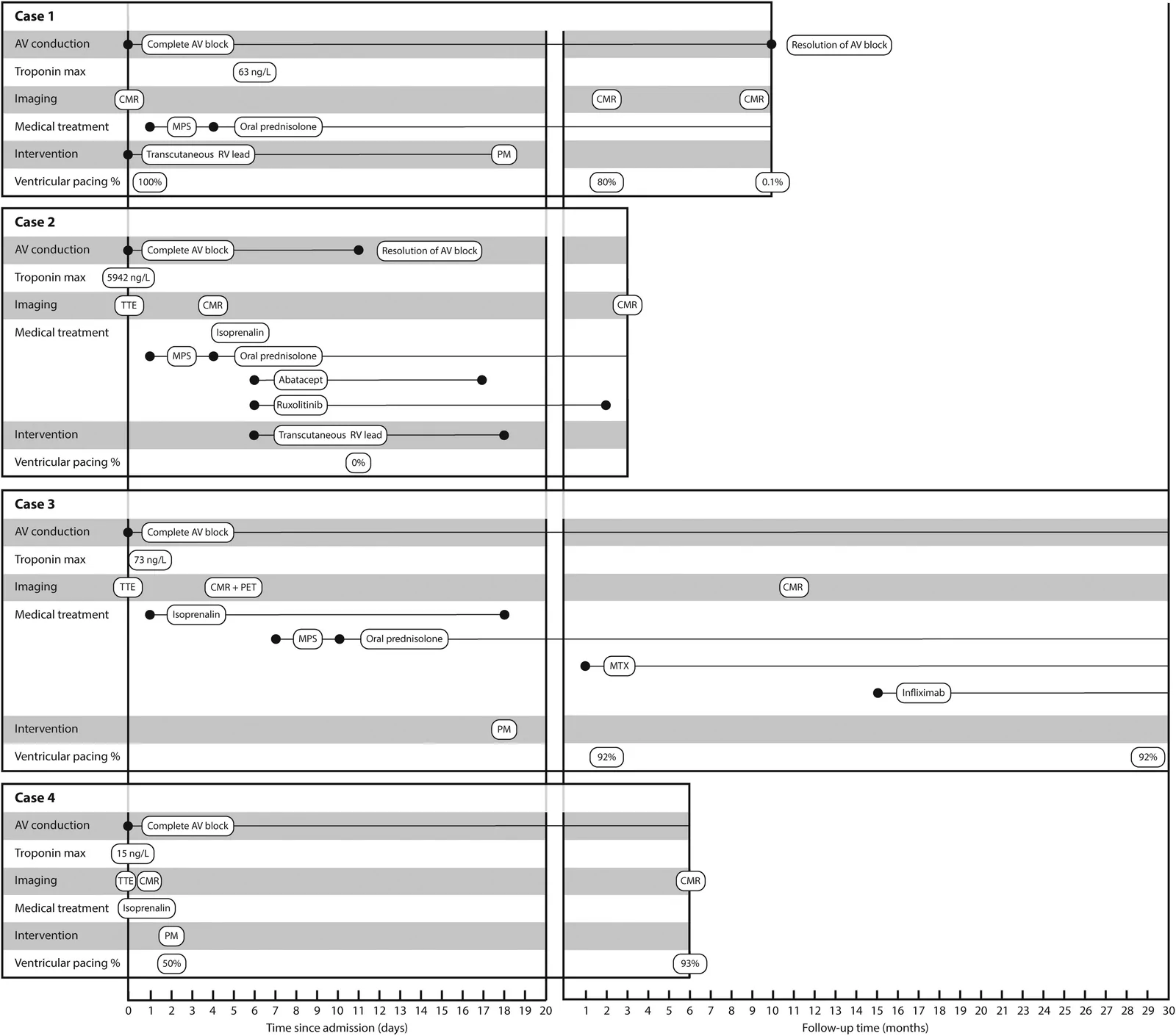
Detailed timeline for the presented cases. CMR, cardiac magnetic resonance; MPS, methylprednisolone; MTX, methotrexate; PET, positron emission tomography; PM, definite pacemaker implantation; TTE, transthoracic echocardiography; Troponin max, maximal troponin levels during admission; RV, right ventricle; other abbreviations as in *Summary Figure*.

Current ESC guidelines recommend consideration of multimodality imaging for myocardial tissue characterization in patients with conduction abnormalities requiring pacemaker implantation, particularly in those younger than 60 years (Class IIa, C).^4^ One potential cause of such conduction abnormalities is myocarditis, in which CMR is recommended not only for initial characterization but also for follow-up (Class I, C).^5^ We present a case series of four patients illustrating the role of CMR in identifying myocardial inflammation of different aetiologies as the underlying cause of advanced conduction abnormalities, with potential implications for subsequent management and device therapy decisions (*Summary Figure*). A detailed timeline of all four cases is presented in *Figure 1*.

## Summary figure

**Figure:**
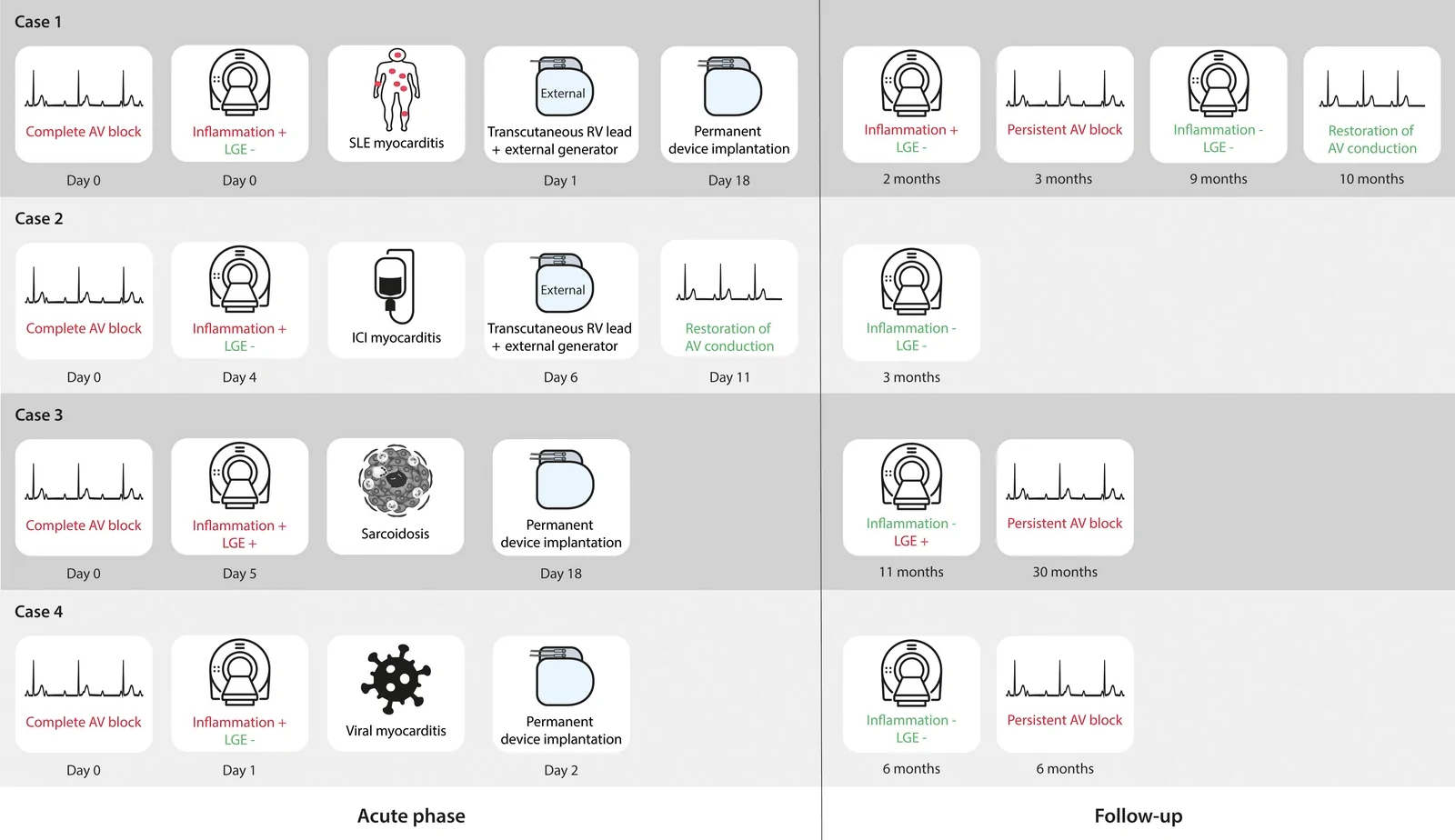
AV, atrioventricular; LGE, late gadolinium enhancement; SLE, systemic lupus erythematosus; ICI, immune checkpoint inhibitor.

## Patient 1

**Figure 2:**
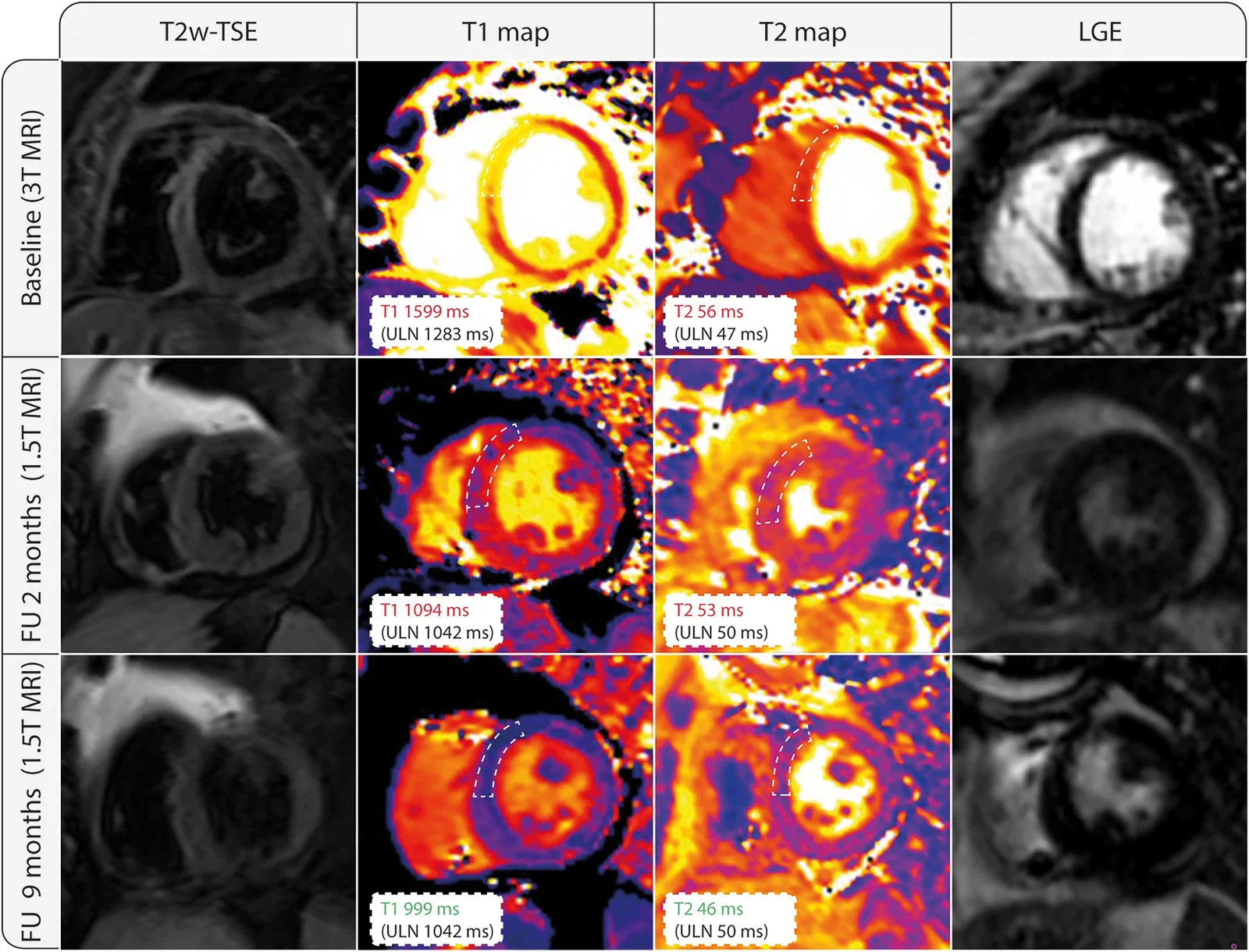
CMR findings in Case 1, a patient with SLE myocarditis. Baseline CMR demonstrated increased signal intensity basal anteroseptal on T2-weighted turbo spin echo (T2w-TSE), globally increased myocardial T1 and T2 values on mapping, anteroseptal mid, respectively, 1599 and 56 ms, and no late gadolinium enhancement (LGE). CMR imaging after 2 months showed persisting albeit decreased myocardial inflammation with normal T2w-TSE images but slightly elevated T1 and T2 values on mapping (1094 and 53 ms anteroseptal mid, respectively) and no LGE. CMR imaging after 9 months demonstrated complete normalization without signs of myocardial inflammation on T2w-TSE and T1 and T2 mapping and absence of LGE. Abbreviations as in *Figure 1*.

A 72-year-old woman with a prior history of SLE and rheumatoid arthritis, treated with methotrexate and hydroxychloroquine, presented with exertional dyspnoea and lightheadedness. Electrocardiogram (ECG) showed sinus rhythm with a prolonged PR interval. Ambulant rhythm monitoring demonstrated episodes of symptomatic complete AV block. High-sensitivity troponin T [hs-troponin T, 63 ng/L; upper limit of normal (ULN) 14 ng/L] and erythrocyte sedimentation rate (ESR, 68 mm/h; ULN 30 mm/h) were slightly elevated, and renal function was mildly decreased [estimated glomerular filtration rate (eGFR): 48 mL/min/1.73 m^2^]. The patient was admitted with initiation of isoprenaline. To evaluate the underlying cause, CMR imaging on a 3-Tesla (3T) scanner (Magnetom Vida, Siemens Healthineers) was performed showing normal left ventricular (LV) and right ventricular (RV) function and absence of LGE (*Figure 2*). However tissue characterization was consistent with myocardial oedema, based on T2w-TSE and increased T1 and T2 mapping, most pronounced in the midventricular anteroseptal wall (T1 1599 ms, Z-score 9.3; T2 56 ms, Z-score 5.5; scanner-specific ULN are provided in the Supplementary data, segmental ULN 1283 and 47 ms, respectively). Based on the Lake–Louise criteria, the diagnosis of myocarditis was made.^5^ Since myocarditis was possibly associated with SLE, high dose intravenous methylprednisolone was initiated, followed by oral prednisolone. Despite treatment, the AV block progressed and patient required pacing. Given the potential reversible cause detected by CMR, an RV lead for permanent use with active fixation was inserted transcutaneously and connected to an external pacemaker generator for temporary pacing. Atrioventricular conduction did not recover during 2 weeks of hospital stay, and a permanent dual-chamber pacemaker was implanted. Serial CMR imaging was performed after 2 and 9 months. Cardiac magnetic resonance imaging after 2 months on a 1.5T-scanner (Magnetom Sola, Siemens Healthineers) was performed to guide tapering of corticosteroid treatment and showed persisting albeit decreased myocardial inflammation with no increased signal intensity anteroseptal on T2w-TSE but slightly elevated T1 (1094 ms, ULN 1042 ms, Z-score 3.9) and T2 values (53 ms, ULN 50 ms, Z-score 3.6) on mapping. After 9 months, CMR showed complete normalization with no LGE and no signs of myocardial inflammation. Strikingly, ventricular pacing gradually decreased in concordance with CMR-defined resolution of inflammation, from 100% in the acute phase to 80% at 2 months and 0.1% at 10 months of follow-up with normal AV conduction.

## Patient 2

**Figure 3:**
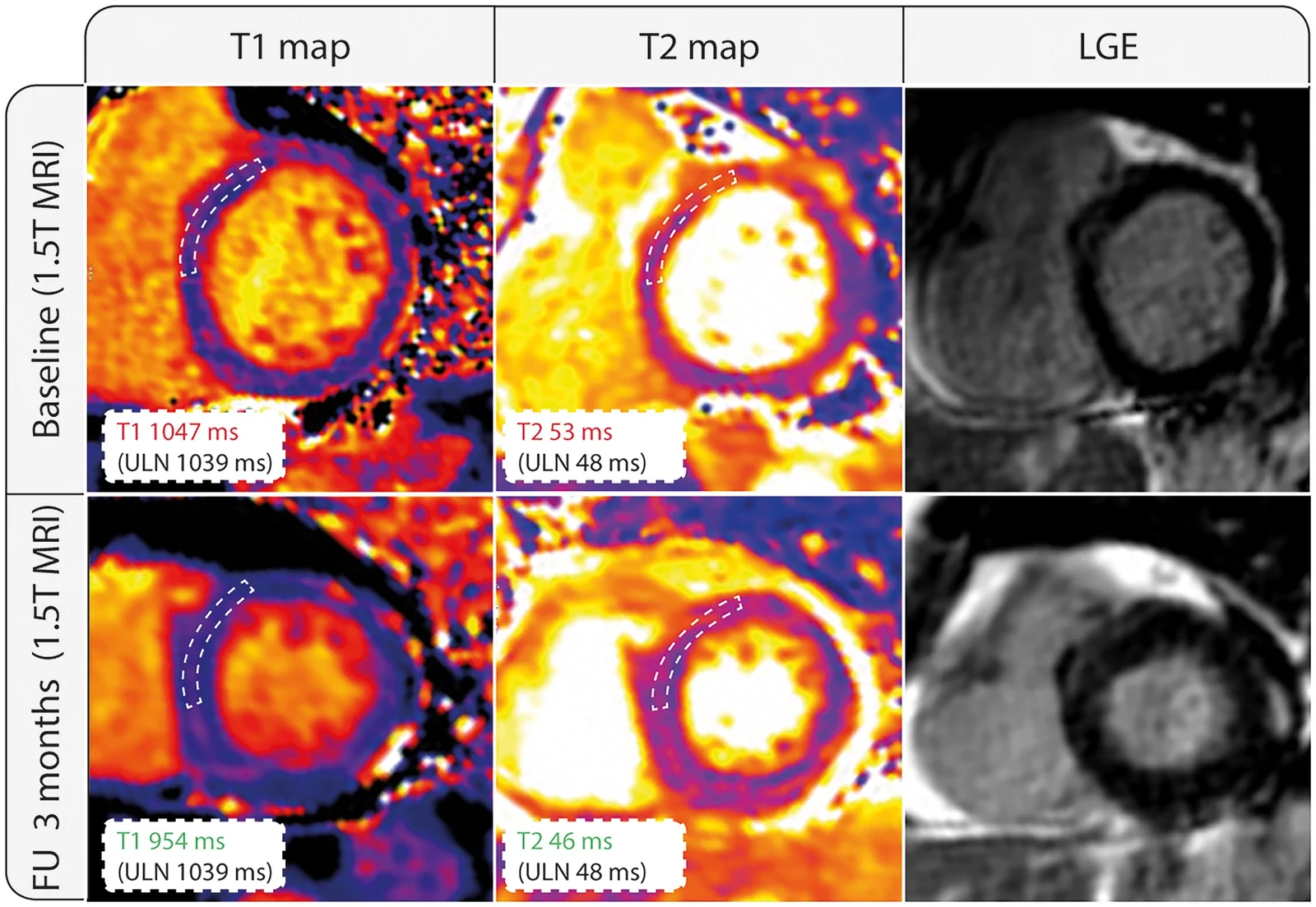
CMR findings in Case 2, a patient with ICI-induced myocarditis. Baseline CMR showing elevated T1 and T2 values anteroseptal (1047 and 53 ms, respectively) and no LGE, taken together consistent with myocarditis. CMR after 3 months demonstrated complete resolution of myocardial inflammation with normalization of myocardial T1 and T2 values and still no LGE. Abbreviations as in *Figure 1*.

A 78-year-old woman with a prior history of non-small cell lung carcinoma for which she had completed chemoradiation and received the second dose of adjuvant durvalumab, an antiProgrammed Cell Death-Ligand 1 ICI, 2 days prior, presented with generalized myalgia and fatigue. Creatine kinase (CK), hs-troponin T, and hs-troponin I levels were markedly elevated (maximal 10322 U/L, 5942 ng/L, and >1000 ng/L, respectively) consistent with a combined durvalumab-induced myositis and myocarditis. Furthermore, ECG showed an asymptomatic complete AV block with adequate nodal escape. Echocardiography demonstrated normal biventricular function. Patient was treated with high dose methylprednisolone followed by oral prednisolone. Cardiac magnetic resonance imaging on a 1.5T-scanner (Magnetom Sola) showed normal LV function without LGE, but T1 and T2 mapping demonstrated myocardial oedema most pronounced in the basal anteroseptal wall (T1 1047 ms, Z-score 2.4, and T2 53 ms, Z-score 5.1), consistent with ICI-myocarditis (*Figure 3*). T2-weighted turbo spin echo was not performed in this patient due to patient discomfort and the need to minimize scan time. Given the development of non-sustained ventricular tachycardia and a second rise in cardiac enzymes indicating steroid refractoriness, the cytotoxic T-lymphocyte-associated protein-4 agonist abatacept and Janus kinase 1/2 inhibitor ruxolitinib were added as second-line immunosuppressants (in accordance with clinical trial protocols NCT05195645 and NCT05335928) on Day 6 of hospital stay. Additionally on Day 6, AV block became symptomatic which persisted despite isoprenaline, and a RV lead with active fixation was placed transcutaneously and connected to an external generator for temporary pacing. Subsequently CK and hs-troponin I markedly decreased to near normal levels (390 U/L and 24 ng/L, respectively) within 6 weeks after starting second-line immunosuppressants and patient became asymptomatic. In parallel, AV conduction recovered, temporary pacemaker could be explanted, and the patient was discharged without a permanent device. Serial MRI after 3 months demonstrated complete resolution of myocardial inflammation (T1 954 ms, Z-score −2, 7 and T2 46 ms, Z-score 0.4) and no LGE.

## Patient 3

**Figure 4:**
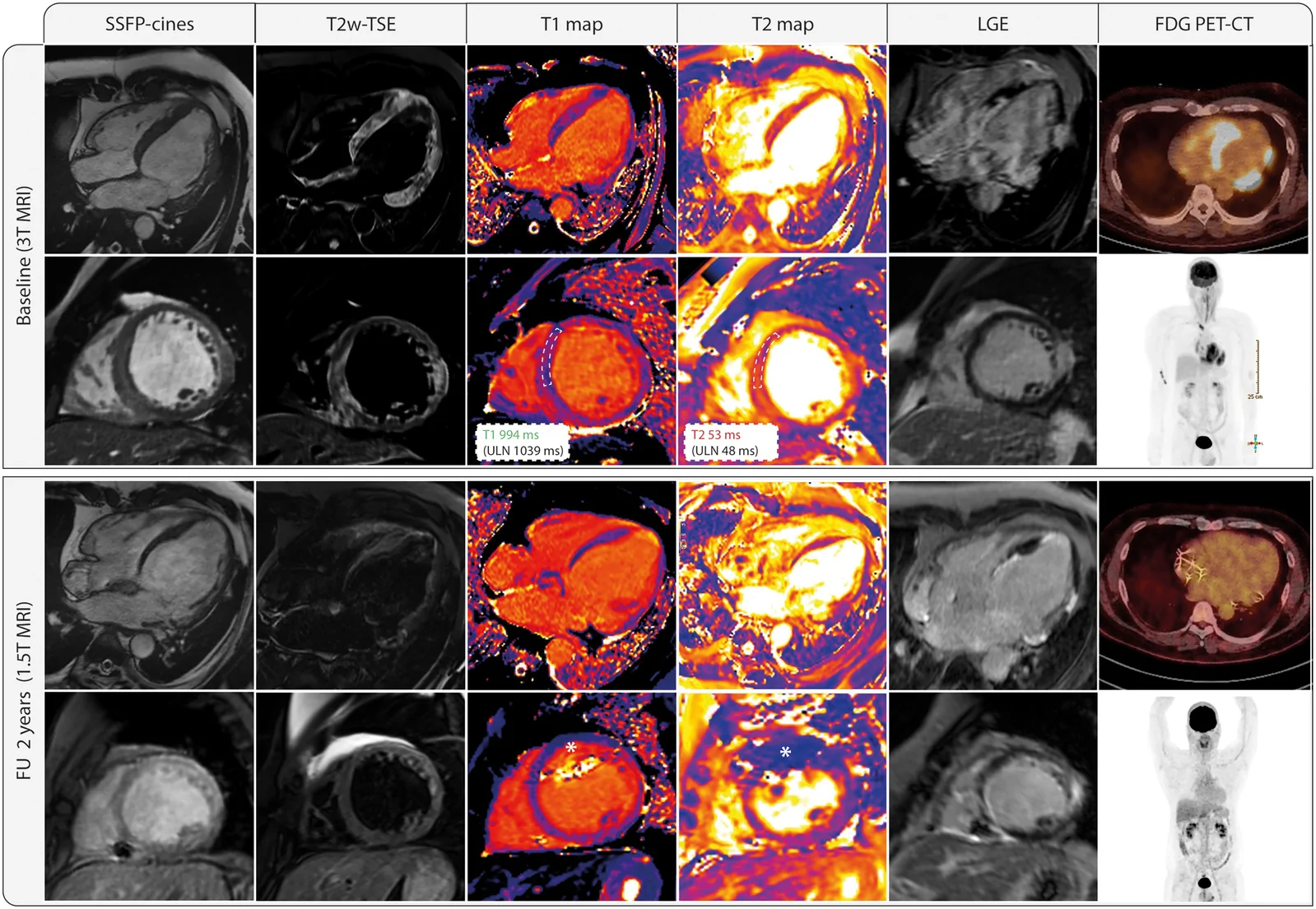
CMR findings in Case 3, a patient with cardiac sarcoidosis. Baseline CMR and fluorodeoxyglucose positron emission tomography computed tomography (FDG PET-CT) imaging: SFFP cine images showing mildly dilated left ventricle (LV) with wall thinning of the basal septum, multifocally increased signal intensity on T2w-TSE, regionally increased myocardial T1 values anterior/inferior (anteroseptal normal T1 values 994 ms) with diffusely increased T2 values (mean 54 ms, anteroseptal 53 ms), and extensive multifocal nearly transmural LGE matching the areas with increased T1 values. Lastly, FDG PET-CT demonstrating increased tracer uptake in the myocardium consistent with sarcoidosis. Serial imaging with CMR (after 11 months) and PET-CT (after 5 months) showed progressive LV dilatation on SFFP cine images; resolution of inflammation as demonstrated by normalization of T2w-TSE images and normalization of myocardial T1 and T2 values, apart from the artefact caused by the CRT-D (marked with an asterisk); and persistent multifocal pattern of LGE in the same regions as previously. Lastly, FDG PET-CT showed reduced disease activity demonstrated by decreased myocardial FDG uptake. Abbreviations as in *Figure 1*.

A 47-year-old man with no prior medical history was admitted after reporting transient loss of consciousness due to intermittent complete AV block with an inadequate escape rhythm and was started on isoprenaline. Echocardiography showed moderately reduced LV function with akinesia and wall thinning of the basal septum. Cardiac magnetic resonance imaging (on a 1.5T-scanner, Magnetom Vida) was performed and showed moderately reduced LV function and multifocal areas of LGE in a nonischaemic distribution (*Figure 4*). Tissue characterization, using T2w-TSE and both T1 and T2 mapping, showed myocardial oedema matching these areas with LGE, including the basal anteroseptal wall (T2 53 ms, Z-score 5.1), meeting the Lake–Louise criteria for myocarditis. Additional 18F-fluorodeoxyglucose positron emission tomography computed tomography (FDG PET-CT) demonstrated increased tracer uptake in the myocardium and mediastinal lymph nodes reinforcing the suspicion of sarcoidosis. Endobronchial ultrasonography with aspiration of mediastinal lymph nodes confirmed the presence of non-necrotizing granulomatous inflammation. Therefore, diagnostic criteria for cardiac sarcoidosis were met, and patient was treated accordingly with high dose methylprednisolone followed by oral prednisolone and methotrexate.^6^ Given the reduced LV function due to cardiac sarcoidosis, a cardiac resynchronization therapy-defibrillator (CRT-D) was implanted. Serial CMR imaging after 11 months showed resolution of myocardial inflammation with persistent multifocal pattern of LGE. Accordingly, complete AV block persisted during 2.5 years of follow-up.

## Patient 4

**Figure 5:**
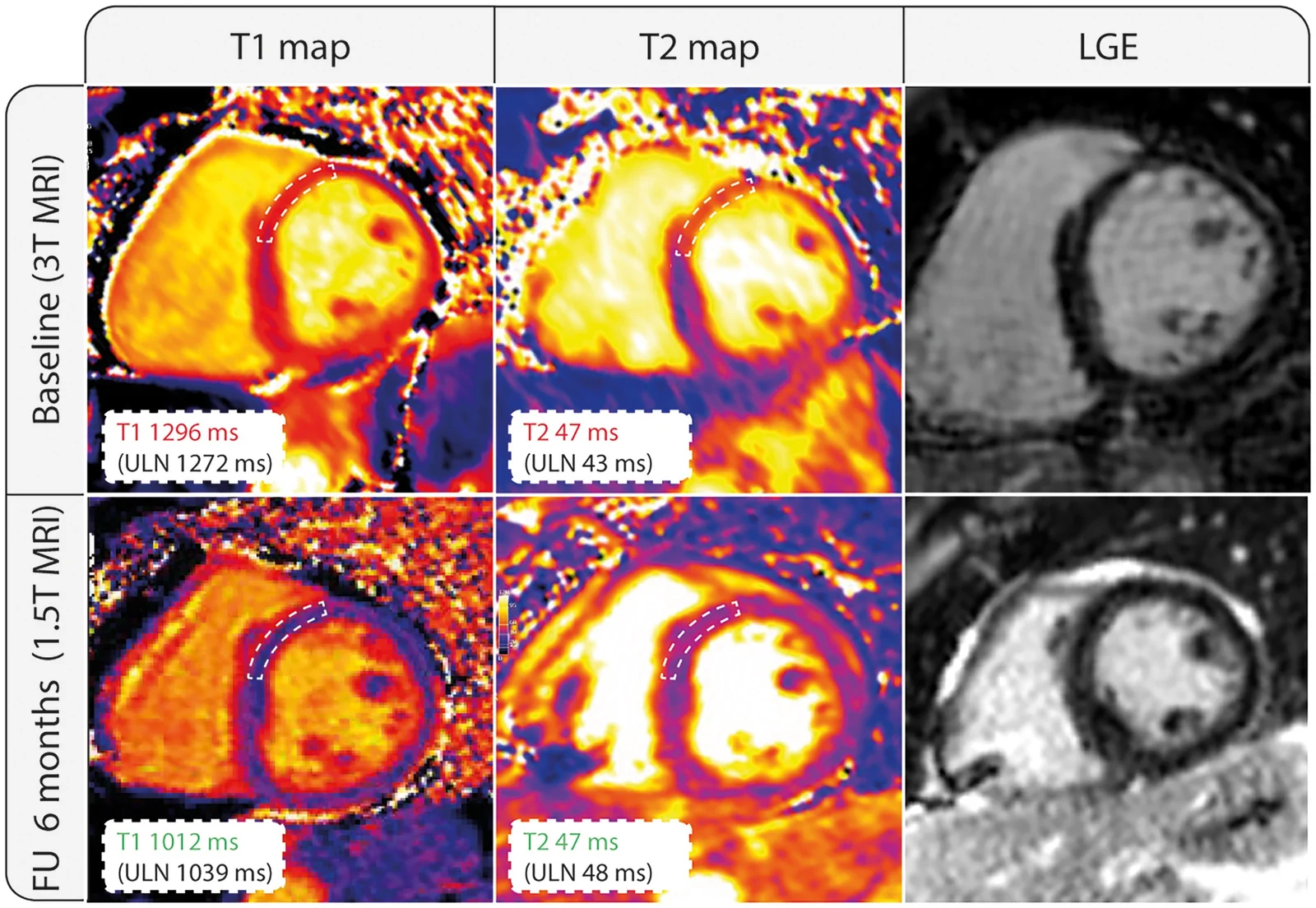
CMR findings in Case 4, a patient with viral myocarditis. Baseline CMR imaging showed increased myocardial T1 and T2 values anteroseptal, anterior, and inferolateral (anteroseptal 1296 and 47 ms, respectively) and no LGE. Follow-up CMR imaging demonstrated complete resolution of myocardial inflammation with normalization of myocardial T1 and T2 values and still no LGE. Abbreviations as in *Figure 1*.

A 53-year-old man without prior cardiovascular disease was admitted because of a symptomatic complete AV block with inadequate ventricular escape for which isoprenaline was initiated. Echocardiography showed normal biventricular function, and hs-troponin T levels were borderline elevated (15 ng/L). Patient reported an episode of coughing 2 weeks prior, and additional lab results showed lymphocytosis, suggestive of a recent viral upper airway infection. Polymerase chain reactions for common viral pathogens were performed but yielded no positive results. Cardiac magnetic resonance imaging on a 3T-scanner (Magnetom Vida) demonstrated normal biventricular function without LGE (*Figure 5*). However, findings with tissue characterization were consistent with myocarditis, i.e. increased signal intensity anteroseptal/anterior on T2w-TSE and increased myocardial T1 and T2 values in the basal anteroseptal, anterior, and inferolateral segments (anteroseptal T1 1296 ms, Z-score 2.7, and T2 47 ms, Z-score 4.3). Given persistent haemodynamic instability due to persistent AV block with inadequate escape rhythm despite isoprenaline treatment, a two-chamber pacemaker was implanted on Day 2 of hospital stay. Additional thoracic CT scan and biomarkers did not demonstrate an underlying systemic auto-immune disease. Therefore, in combination with the history suggestive of a recent viral infection, myocarditis was deemed to have been caused by a post-viral immune response. However, the possibility of isolated conduction system inflammation or another inflammatory/conduction disorder phenotype could not be completely ruled out. During 6-months of follow-up, complete AV block persisted with a ventricular pacing rate of 98%. Surprisingly, simultaneous CMR imaging demonstrated complete resolution of myocardial inflammation with normalization of myocardial T1 and T2 values (T1 1012 ms, Z-score 0.5, and T2 47 ms, Z-score 1.1) and still no LGE.

## Discussion

Atrioventricular conduction disorders are a rare complication of acute myocarditis, occurring in only 1–2% of cases.^2,3^ Inflammation of the conduction system is thought to temporarily impair AV conduction with resolution of conduction once inflammation is resolved.^7,8^ However, numerous studies have reported persisting AV block after myocarditis,^9–12^ demonstrating the variable course of AV conduction disorders in the context of myocarditis. The aetiology and associated underlying immune phenomena appear to influence reversibility. Specifically in cardiac sarcoidosis, recovery of AV conduction may be influenced by immunosuppressants; however, long term recovery rate is unpredictable and relatively low.^10,12,13^ High recovery rates have been reported in viral or ICI-associated myocarditis,^7,8^ whereas limited data exists on the recovery of AV conduction in SLE myocarditis.

The present series reflects the full spectrum of AV conduction recovery (*Summary Figure*) and provides insight into potential pathophysiological mechanisms as assessed by serial CMR. Three clinical scenarios can be distinguished. First, transient myocardial inflammation may lead to reversible AV block, with restoration of conduction paralleling resolution of inflammation on CMR (Cases 1 and 2). Second, inflammation may result in irreversible myocardial injury and fibrosis, visualized as LGE, leading to permanent AV block despite resolution of acute inflammation (Case 3). Third, persistent AV block may occur despite resolution of inflammation and absence of LGE on CMR (Case 4), as previously described in cases of viral and eosinophilic myocarditis.^9,14^ The underlying pathophysiological process in this last scenario is not clearly understood. Persisting AV conduction disorders may be caused by diffuse myocardial fibrosis in the conduction system, which can be missed due to pacemaker artefacts resulting in underestimation of T1 values on mapping.^15^ Furthermore, given the extremely delicate structure of the conduction system, minor changes that are functionally insignificant to the myocardium could impair the conducting pathway and cause functional impairment of AV conduction.^14^ These minor changes may be missed by all conventional CMR techniques. Additional electrophysiology study for the localization of the AV block, i.e. nodal vs. infranodal, may provide additional prognostic information in these patients.^4^

**Figure 6:**
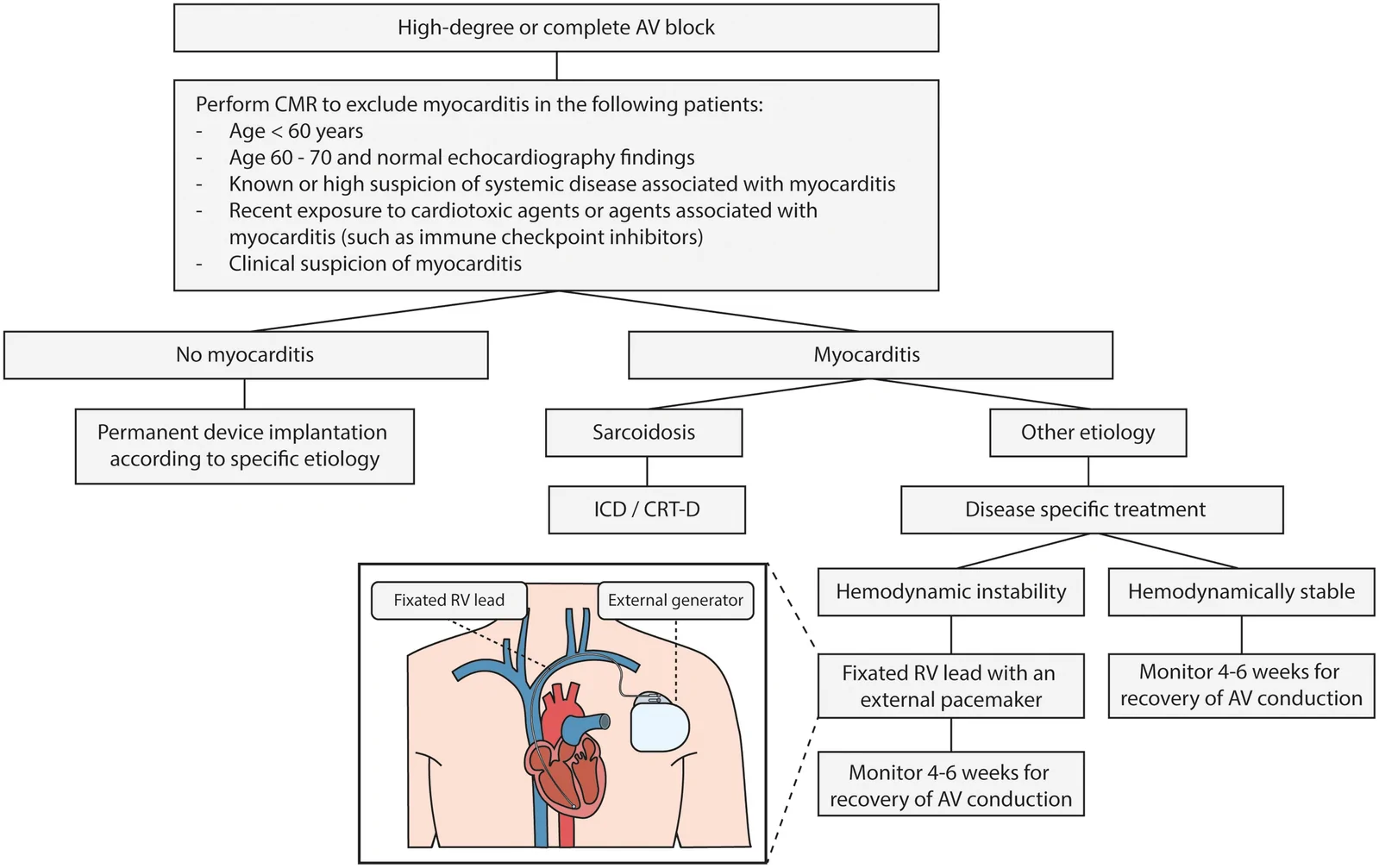
Proposed expert opinion based strategy for the work-up and treatment of patients with high-degree or complete AV block. CRT-D: cardiac resynchronization therapy-defibrillator; ICD, implantable cardioverter–defibrillator; other abbreviations as in *Figure 1*.

To systematically approach and treat patients with AV block in myocarditis, we propose the expert opinion based strategy as illustrated in *Figure 6*. In accordance with the ESC guidelines, CMR should be considered in all patients below 60 years of age to evaluate the presence of myocarditis (Class IIa, C).^4,5^ Additionally, we propose to consider CMR in 1) patients aged 60–70 years with normal echocardiography findings, 2) patients with a known or high suspicion of systemic disease associated with myocarditis, 3) patients with recent exposure to cardiotoxic agents or agents associated with myocarditis (such as ICI), and 4) patients with a clinical suspicion of myocarditis, regardless of age. When myocarditis is excluded by CMR, device implantation should follow standard indications according to the underlying aetiology and ventricular function. Since acute myocarditis due to ICI may be missed by CMR, clinicians should be cautious to rule out myocarditis based on normal CMR findings in patients receiving ICI and high clinical suspicion.^16^ When CMR confirms the diagnosis of myocarditis, it is important to exclude sarcoidosis as underlying aetiology, given the unpredictable recovery of conduction^10,12^ and the increased risk of sudden cardiac death in these patients.^17,18^ Current ESC guidelines therefore advise to implant a ICD or CRT-D (depending on LV function) rather than a pacemaker in cardiac sarcoidosis (Class IIa, C).^4^

In non-sarcoid myocarditis, disease specific treatment should be initiated, such as immunosuppressive medication for SLE or ICI-myocarditis and watchful waiting for mild viral myocarditis. Given the often transient nature of AV conduction disorders, current ESC guidelines advise to wait for potential recovery before definite device implantation.^4,5^ We propose the implantation of a transcutaneous RV lead with active fixation connected to an external generator for temporary pacing, as in Cases 1 and 2 and as recently reported in a series of ICI-induced myocarditis.^8^ In contrast with a balloon-tipped transvenous pacing catheter which can only stay in place for a short period of time (i.e. days), a transcutaneous RV lead can be sutured to the skin which allows for weeks of temporary pacing in which patients retain normal mobility. This approach may lower complication rate and may be more comfortable for patients; however, given the presence of an external generator, hospitalization remains standard practice in our institution. If AV conduction recovers, the temporary pacemaker may be removed. In persisting AV block after a period of 4–6 weeks, depending on the clinical likelihood of recovery, a definite device must be implanted.

The ESC guidelines advise to perform serial CMR in all patient within the first 6 months to evaluate disease evolution (Class I, C).^4^ Cardiac magnetic resonance-defined resolution of myocardial inflammation has been associated with an increase in LV function and a favourable prognosis, especially in the absence of residual fibrosis as determined by LGE, which may disappear during follow-up.^19,20^ A previous study reported complete recovery of myocarditis on follow-up CMR in all eight patients presenting with myocardial oedema without baseline LGE, suggesting that isolated oedema carries a favourable prognosis.^19^ However, the prognostic value of baseline imaging and the impact of its resolution on recovery specifically from complete AV block remain largely unstudied, particularly in myocarditis-associated cases. This case series offers preliminary observations on baseline imaging and AV block resolution, prompting the need for larger-scale investigations into these potential predictors.

The interpretation of the described cases and the proposed clinical strategy are hampered by several limitations. First, this case series constitutes four typical cases that together with our clinical experience informed the proposed strategy. The proposed strategy is therefore extrapolated from clinical guidelines, anecdotal evidence, and clinical experience and should be viewed as expert opinion. The strategy has been evolving in the last few years, and in retrospect in Case 4, permanent device implantation could have been postponed to wait for potential recovery. Second, myocardial inflammation can occur in a variety of diseases, and therefore one must be cautious to extrapolate the expected disease course and conduction recovery across different aetiologies of inflammatory myocardial disease. Third, longitudinal disease monitoring with CMR was hampered in the current Case 1 and 4 due to the fact that different CMR scanners were used at baseline and follow-up, i.e. 3T and 1.5T inherent to the presence of a cardiac device at follow-up. Nevertheless, for both field strengths, abnormal T1 and T2 values were defined as exceeding 2 standard deviations above local reference values specific to vendor and field strength. These reference values were established in healthy volunteers at the initiation of scanner use (see Supplementary material online, *Tables S1*–*S4*), and regular phantom testing is performed to verify the accuracy and consistency of the measured values. Furthermore, segmental Z-scores were calculated and provided which has been shown to remove the variation of quantitative mapping results across different MRI systems and field strengths.^21^ Fourth, CMR imaging in patients with intracardiac devices is hampered by several potential artefacts due to off-resonance and field inhomogeneity, leading to underestimation of myocardial T1 values on mapping.^15^ Fifth, according to the ESC guideline, the updated Lake–Louise criteria were used for the diagnosis of myocarditis.^5^ Despite the fact that these criteria were met in all four presented cases, we acknowledge the diagnostic uncertainty in Case 4 given the only mildly elevated troponin levels, the absence of LGE, and the persistent AV block despite resolution of inflammation during follow-up CMR. Last, the four presented cases represent biologically heterogeneous inflammatory diseases with potentially different mechanisms of conduction system injury and recovery, potentially limiting the generalizability of the proposed clinical strategy.

In conclusion, AV conduction disorders are a rare complication of acute myocarditis with a heterogeneous and sometimes unpredictable course. Although often transient in nature, persistent AV block may occur, especially in cardiac sarcoidosis. Cardiac magnetic resonance imaging is critical in identifying underlying myocarditis in at-risk patients, can be used as an adjunctive tool for disease monitoring, and may contribute to clinical decision-making regarding device therapy. However, discrepancies between CMR findings and conduction recovery highlight the need for further research into the mechanisms and predictors of persistent conduction disease.

## Lead author biography


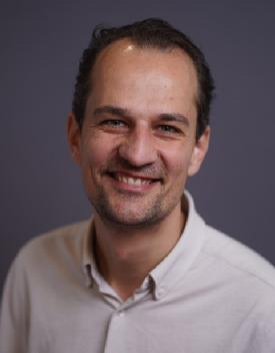
Michiel Bom is a cardiology resident at the Amsterdam UMC, Amsterdam, the Netherlands. He has a strong interest in cardiovascular imaging, specializing in cardiac MR and CT.

## Supplementary Material

ytag699_Supplementary_Data

## Data Availability

No new data were generated or analysed in support of this research.

## References

[1] Ammirati E, Moslehi JJ. Diagnosis and treatment of acute myocarditis: a review. JAMA 2023;329:1098–1113. 10.1001/jama.2023.3371 37014337

[2] Ammirati E, Cipriani M, Moro C, Raineri C, Pini D, Sormani P, et al Clinical presentation and outcome in a contemporary cohort of patients with acute myocarditis: multicenter Lombardy Registry. Circulation 2018;138:1088–1099. 10.1161/CIRCULATIONAHA.118.035319 29764898

[3] Ogunbayo GO, Elayi SC, Ha LD, Olorunfemi O, Elbadawi A, Saheed D, et al Outcomes of heart block in myocarditis: a review of 31,760 patients. Heart Lung Circ 2019;28:272–276. 10.1016/j.hlc.2017.12.005 29402690

[4] Glikson M, Nielsen JC, Kronborg MB, Michowitz Y, Auricchio A, Barbash IM, et al 2021 ESC guidelines on cardiac pacing and cardiac resynchronization therapy. Eur Heart J 2021;42:3427–3520. 10.1093/eurheartj/ehab364 34455430

[5] Schulz-Menger J, Collini V, Groschel J, Adler Y, Brucato A, Christian V, et al 2025 ESC guidelines for the management of myocarditis and pericarditis. Eur Heart J 2025;46:3952–4041. 10.1093/eurheartj/ehaf192 40878297

[6] Cheng RK, Kittleson MM, Beavers CJ, Birnie DH, Blankstein R, Bravo PE, et al Diagnosis and management of cardiac sarcoidosis: a scientific statement from the American Heart Association. Circulation 2024;149:e1197–e1216. 10.1161/CIR.0000000000001240 38634276

[7] Miyake CY, Teele SA, Chen L, Motonaga KS, Dubin AM, Balasubramanian S, et al In-hospital arrhythmia development and outcomes in pediatric patients with acute myocarditis. Am J Cardiol 2014;113:535–540. 10.1016/j.amjcard.2013.10.021 24332245

[8] Palaskas NL, King NE, Ostos-Mendoza KC, Ruiz-Jurado A, Ali HJ, Koutroumpakis E, et al Pacing solutions for immune checkpoint myocarditis and associated conduction disorders. JACC Case Rep 2025;30:103187. 10.1016/j.jaccas.2024.103187 PMC12046784 40250924

[9] Messner M, Mayr A, Zaruba MM, Poelzl G. Eosinophilic myocarditis complicated by permanent atrioventricular nodal block: a case report. Eur Heart J Case Rep 2022;6:ytac055. 10.1093/ehjcr/ytac055 PMC8922707 35295727

[10] Austin BC, Sykora D, Rosenbaum AN, Cooper LT, Gomez EM, Ezzeddine OA, et al Management and outcomes of cardiac sarcoidosis diagnosed after permanent pacemaker implantation for high-grade atrioventricular block. Heart Rhythm 2025;23:e875–e876. 10.1016/j.hrthm.2025.07.045 40752878

[11] Asano K, Noguchi M, Amemiya K, Ikeda Y, Obunai K. Chronic active myocarditis presenting as an isolated atrioventricular block: a case report. Eur Heart J Case Rep 2025;9:ytaf454. 10.1093/ehjcr/ytaf454 PMC12495037 41050527

[12] Sadek MM, Yung D, Birnie DH, Beanlands RS, Nery PB. Corticosteroid therapy for cardiac sarcoidosis: a systematic review. Can J Cardiol 2013;29:1034–1041. 10.1016/j.cjca.2013.02.004 23623644

[13] Kandolin R, Lehtonen J, Kupari M. Cardiac sarcoidosis and giant cell myocarditis as causes of atrioventricular block in young and middle-aged adults. Circ Arrhythm Electrophysiol 2011;4:303–309. 10.1161/CIRCEP.110.959254 21427276

[14] Tashiro K, Kashimura T, Sakai R, Inomata T. Permanent pacemaker timing dilemma in recovered lymphocytic myocarditis. JACC Case Rep 2026;31:106239. 10.1016/j.jaccas.2025.106239 PMC12926214 41335058

[15] Fenski M, Groschel J, Gatehouse P, Kolbitsch C, Schulz-Menger J. Artifacts in cardiac T1 and T2 mapping techniques-influence on reliable quantification. J Cardiovasc Magn Reson 2025;27:101934. 10.1016/j.jocmr.2025.101934 PMC12670910 40721019

[16] Driessen RS, Biesbroek PS, van Rosendael PJ, Slaats J, Eijsvogels TMH, Selder JL, et al Immune checkpoint inhibitor myocarditis: when profound troponin elevation diverges from unremarkable imaging. ESC Heart Fail 2026;13:xvag029. 10.1093/eschf/xvag029 PMC13036827 41711249

[17] Kron J, Sauer W, Schuller J, Bogun F, Crawford T, Sarsam S, et al Efficacy and safety of implantable cardiac defibrillators for treatment of ventricular arrhythmias in patients with cardiac sarcoidosis. Europace 2013;15:347–354. 10.1093/europace/eus316 23002195

[18] Schuller JL, Zipse M, Crawford T, Bogun F, Beshai J, Patel AR, et al Implantable cardioverter defibrillator therapy in patients with cardiac sarcoidosis. J Cardiovasc Electrophysiol 2012;23:925–929. 10.1111/j.1540-8167.2012.02350.x 22812589

[19] Aquaro GD, Ghebru Habtemicael Y, Camastra G, Monti L, Dellegrottaglie S, Moro C, et al Prognostic value of repeating cardiac magnetic resonance in patients with acute myocarditis. J Am Coll Cardiol 2019;74:2439–2448. 10.1016/j.jacc.2019.08.1061 31727281

[20] Vermes E, Childs H, Faris P, Friedrich MG. Predictive value of CMR criteria for LV functional improvement in patients with acute myocarditis. Eur Heart J Cardiovasc Imaging 2014;15:1140–1144. 10.1093/ehjci/jeu099 24925145

[21] Razzaq S, Haririsanati L, Eyre K, Garg R, Chetrit M, Friedrich MG. Inter-scanner comparability of Z-scores for native myocardial T1 and T2 mapping. J Cardiovasc Magn Reson 2024;26:100004. 10.1016/j.jocmr.2023.100004 PMC11211228 38211657

