# Supplementary material for: The many faces of atrioventricular block in myocarditis: a case series and proposed clinical strategy highlighting cardiovascular magnetic resonance-guided diagnosis, device selection, and disease monitoring
Source: Eur Heart J Case Rep. 2026 Sep 15;10(9):ytag699. doi: 10.1093/ehjcr/ytag699 (PMC13614263; doi:10.1093/ehjcr/ytag699)
Supplement: Supplementary file 1 [file ytag699_supplementary_data.docx]

**Supplementary Data**

**Supplementary Methods**

**CMR acquisition protocol**

CMR was performed on a clinical 1.5T or 3T MR system (respectively Magnetom Sola or Vida; Siemens Healthineers, Erlangen, Germany). Cardiac function was assessed using balanced steady-state free precession cine imaging in four-, three-, and two- chamber long-axis and short-axis orientations covering the entire LV from the mitral valve plane to the apex.

T1 mapping was performed using a Modified Look-Locker Inversion Recovery (MOLLI) pulse sequence. T2 mapping was performed using a T2-prepared SSFP images, with three different T2 preparation times. Both T1 and T2 maps were performed within a breath-hold and obtained at the basal and midventricular level. T1 and T2 mapping of the apical segments is not routinely performed at our institution because of the known partial volume effects of the thin wall and susceptibility to breathing or cardiac motion artifacts. LGE images were obtained 12–15 min after administration of a gadolinium-based contrast agent (0.15 mmol/kg, Dotarem®, Guerbet, Roissy, France) using a phase-sensitive segmented inversion recovery gradient-echo pulse sequence. Slice positions of the LGE images were identical to those of the cine images.

**Parametric mapping analysis and local references values**

Abnormal T1 and T2 values were defined as exceeding 2 standard deviations above local reference values specific to vendor and field strength. These reference values were established in healthy volunteers at the initiation of scanner use (**Table S1-S4**). For 1.5T, 29 subjects without prior cardiovascular disease (mean age 33 years, 16 male and 13 female) underwent T1 mapping and 24 subjects (mean age 33 years, 14 male and 10 female) underwent T2 mapping. For 3T, 26 subjects (mean age 34 years, 14 male and 12 female) underwent T1 mapping and 26 subjects (mean age 34 years, 14 male and 12 female) underwent T2 mapping. Scanner- and field strength–specific reference values derived from healthy volunteers were used to calculate z-scores per segment for each patient, as previously described:

z-score = (patient T1/T2 value − mean reference T1/T2 value) ÷ standard deviation of the reference T1/T2 values.^(1)^

**Supplemental references**

1.	Razzaq S, Haririsanati L, Eyre K, Garg R, Chetrit M, Friedrich MG. Inter-scanner comparability of Z-scores for native myocardial T1 and T2 mapping. J Cardiovasc Magn Reson. 2024;26:100004.

**Table S1.** Local segmental reference values for T1 mapping on 1.5 T (Magnetom Sola)

|  | T1 values | | |
| --- | --- | --- | --- |
|  | Mean ± SD | LLN | ULN |
| Segment 1 (n=27) | 980 ± 24 | 932 | 1028 |
| Segment 2 (n=28) | 1003 ± 18 | 967 | 1039 |
| Segment 3 (n=26) | 1004 ± 24 | 956 | 1052 |
| Segment 4 (n=25) | 999 ± 18 | 963 | 1035 |
| Segment 5 (n=27) | 977 ± 21 | 935 | 1019 |
| Segment 6 (n=27) | 971 ± 20 | 931 | 1011 |
| Segment 7 (n=27) | 969 ± 30 | 909 | 1029 |
| Segment 8 (n=29) | 986 ± 28 | 930 | 1042 |
| Segment 9 (n=27) | 1006 ± 25 | 956 | 1056 |
| Segment 10 (n=26) | 1001 ± 26 | 949 | 1053 |
| Segment 11 (n=28) | 974 ± 24 | 926 | 1022 |
| Segment 12 (n=27) | 980 ± 24 | 932 | 1028 |

SD: standard deviation; LLN: lower limit of normal = mean – 2SD; ULN: upper limit of normal = mean + 2SD. Segment numbering according to AHA 17-segment model (Figure S1)

**Table S2.** Local segmental reference values for T2 mapping on 1.5 T (Magnetom Sola)

|  | T2 values | | |
| --- | --- | --- | --- |
|  | Mean ± SD | LLN | ULN |
| Segment 1 (n=23) | 45.7 ± 1.8 | 42.1 | 49.3 |
| Segment 2 (n=24) | 45.4 ± 1.5 | 42.4 | 48.4 |
| Segment 3 (n=24) | 44.5 ± 1.5 | 41.5 | 47.5 |
| Segment 4 (n=23) | 45.8 ± 1.9 | 42.0 | 49.6 |
| Segment 5 (n=23) | 45.0 ± 2.2 | 40.6 | 49.4 |
| Segment 6 (n=24) | 45.0 ± 1.5 | 42.0 | 48.0 |
| Segment 7 (n=21) | 46.2 ± 2.0 | 42.2 | 50.2 |
| Segment 8 (n=19) | 46.9 ± 1.7 | 43.5 | 50.3 |
| Segment 9 (n=23) | 45.4 ± 1.7 | 42.0 | 48.8 |
| Segment 10 (n=22) | 45.6 ± 1.6 | 42.4 | 48.8 |
| Segment 11 (n=21) | 45.4 ± 1.7 | 42.0 | 48.8 |
| Segment 12 (n=21) | 45.7 ± 1.7 | 42.3 | 49.1 |

SD: standard deviation; LLN: lower limit of normal = mean – 2SD; ULN: upper limit of normal = mean + 2SD. Segment numbering according to AHA 17-segment model (Figure S1)

**Table S3.** Local segmental reference values for T1 mapping on 3 T (Magnetom Vida)

|  | T1 values | | |
| --- | --- | --- | --- |
|  | Mean ± SD | LLN | ULN |
| Segment 1 (n=26) | 1177 ± 44 | 1089 | 1265 |
| Segment 2 (n=25) | 1204 ± 34 | 1136 | 1272 |
| Segment 3 (n=25) | 1214 ± 31 | 1152 | 1276 |
| Segment 4 (n=23) | 1211 ± 42 | 1127 | 1295 |
| Segment 5 (n=26) | 1185 ± 41 | 1103 | 1267 |
| Segment 6 (n=26) | 1176 ± 42 | 1092 | 1260 |
| Segment 7 (n=26) | 1164 ± 56 | 1052 | 1276 |
| Segment 8 (n=26) | 1197 ± 43 | 1111 | 1283 |
| Segment 9 (n=26) | 1211 ± 45 | 1125 | 1301 |
| Segment 10 (n=25) | 1199 ± 46 | 1107 | 1291 |
| Segment 11 (n=26) | 1173 ± 39 | 1095 | 1251 |
| Segment 12 (n=26) | 1176 ± 42 | 1092 | 1260 |

SD: standard deviation; LLN: lower limit of normal = mean – 2SD; ULN: upper limit of normal = mean + 2SD. Segment numbering according to AHA 17-segment model (Figure S1)

**Table S4.** Local segmental reference values for T2 mapping on 3 T (Magnetom Vida)

|  | T2 values | | |
| --- | --- | --- | --- |
|  | Mean ± SD | LLN | ULN |
| Segment 1 (n=26) | 38.8 ± 2.2 | 34.4 | 43.2 |
| Segment 2 (n=26) | 39.6 ± 1.7 | 36.2 | 43 |
| Segment 3 (n=26) | 38.0 ± 1.3 | 35.4 | 40.6 |
| Segment 4 (n=26) | 38.7 ± 2.1 | 34.5 | 42.9 |
| Segment 5 (n=26) | 39.8 ± 2.9 | 34.0 | 45.6 |
| Segment 6 (n=26) | 38.1 ± 1.6 | 34.9 | 41.3 |
| Segment 7 (n=26) | 40.6 ± 3.1 | 34.4 | 46.8 |
| Segment 8 (n=26) | 41.8 ± 2.6 | 36.6 | 47.0 |
| Segment 9 (n=26) | 37.9 ± 1.7 | 34.5 | 41.0 |
| Segment 10 (n=26) | 37.5 ± 2.2 | 33.1 | 41.9 |
| Segment 11 (n=25) | 40.5 ± 2.95 | 37.4 | 44.6 |
| Segment 12 (n=26) | 38.7 ± 2.0 | 34.7 | 42.7 |

SD: standard deviation; LLN: lower limit of normal = mean – 2SD; ULN: upper limit of normal = mean + 2SD. Segment numbering according to AHA 17-segment model (Figure S1)

**Figure S1. AHA 17-segment model**
